# Sociogenomics of self vs. non-self cooperation during development of *Dictyostelium discoideum*

**DOI:** 10.1186/1471-2164-15-616

**Published:** 2014-07-21

**Authors:** Si I Li, Neil J Buttery, Christopher RL Thompson, Michael D Purugganan

**Affiliations:** Department of Biology, Center for Genomics and Systems Biology, New York University, New York, NY 10003 USA; Faculty of Life Sciences, University of Manchester, Manchester, M13 9PT UK

**Keywords:** Social cooperation, Chimera, Multicellularity, Transcriptome

## Abstract

**Background:**

*Dictyostelium discoideum*, a microbial model for social evolution, is known to distinguish self from non-self and show genotype-dependent behavior during chimeric development. Aside from a small number of cell-cell recognition genes, however, little is known about the genetic basis of self/non-self recognition in this species. Based on the key hypothesis that there should be differential expression of genes if *D. discoideum* cells were interacting with non-clone mates, we performed transcriptomic profiling study in this species during clonal vs. chimeric development. The transcriptomic profiles of *D. discoideum* cells in clones vs. different chimeras were compared at five different developmental stages using a customized microarray. Effects of chimerism on global transcriptional patterns associated with social interactions were observed.

**Results:**

We find 1,759 genes significantly different between chimera and clone, 1,144 genes associated significant strain differences, and 6,586 genes developmentally regulated over time. Principal component analysis showed a small amount of the transcriptional variance to chimerism-related factors (Chimerism: 0.18%, Chimerism × Timepoint: 0.03%). There are 162 genes specifically regulated under chimeric development, with continuous small differences between chimera vs. clone over development. Almost 60% of chimera-associated differential genes were differentially expressed at the 4 h aggregate stage, which corresponds to the initial transition of *D. discoideum* from solitary life to a multicellular phase.

**Conclusions:**

A relatively small proportion of over-all variation in gene expression is explained by differences between chimeric and clonal development. The relatively small modifications in gene expression associated with chimerism is compatible with the high level of cooperation observed among different strains of *D. discoideum*; cells of distinct genetic backgrounds will co-aggregate indiscriminately and co-develop into fruiting bodies. Chimeric development may involve re-programming of the transcriptome through small modifications of the developmental genetic network, which may also indicate that response to social interaction involves many genes with individually small transcriptional effect.

**Electronic supplementary material:**

The online version of this article (doi:10.1186/1471-2164-15-616) contains supplementary material, which is available to authorized users.

## Background

Conflict and cooperation between individuals within social groups have been subjects of intense study in the last few decades [1, 2]. From these studies it has become clear that a key evolutionary innovation is the ability of individuals to discern self from non-self. One way such recognition can be used is to allow individuals to preferentially direct cooperative acts towards genetically related individuals [1, 2], thus permitting them to form kin groups. Such behavior can promote social cooperation. In contrast, however, such recognition can also be used by selfish individuals to increase fitness, by ensuring that they only perform antagonistic acts towards unrelated individuals.

Discrimination between interacting individuals requires a recognition system that is capable of distinguishing genotypes. Such recognition systems have evolved in diverse contexts, including self-incompatibility mechanisms on flowering plants [3], immune defense systems [4], and in nest-mate cooperation in social insects [5]. Understanding the molecular nature of kin recognition as well as subsequent cellular and behavioral consequences is thus a central issue in studies of conflict and the evolution of social cooperation

In the last few years, the social amoeba *Dictyostelium discoideum* has emerged as one of the best model systems for the study of conflict and cooperation in social systems [6]. *D. discoideum* is a eukaryotic microbe that has been a model system for the study of cell-cell interactions, signaling and differentiation [7]. The amoeba generally exist as single-celled individuals, but upon starvation enter a multicellular phase [8]. Up to 10^5^ cells can aggregate, first forming a mound, then a motile slug and eventually developing into a fruiting body. The fruiting body consists of a viable sorus sitting on top of a stalk; the sorus harbors spores that await dispersal and germination when conditions are favorable for vegetative growth, while cells contributing to the stalk undergo cell death during the development of the fruiting body. The aggregation of individual cells to form multicellular fruiting bodies is a clear example of social cooperation [6, 9]. Moreover, the dramatic difference in cell fate during development also gives rise to social conflicts, since stalk cells are sacrificed to allow the survival of sorus cells at the top of the fruiting body.

There is now good evidence supporting the idea that *Dictyosteium* cells can distinguish self from non-self. For example, genetically and geographically distant isolates can recognize and segregate from each other, thus increasing relatedness and reducing the potential for social conflict [10, 11]. The molecular basis of this is now beginning to emerge and is thought to be dependent on polymorphic self-recognition molecules encoded by *tgrB1* and *tgrC1* [12, 13]. Chimerism during fruiting body development, however, frequently occurs. For example, *D. discoideum* strains that are found in close proximity in populations in North America will readily form chimeras with no measurable segregation [14]. Moreover, these strains have been shown to exhibit a social dominance hierarchy in which some strains become overrepresented in the spore population of chimeric fruiting bodies.

Buttery *et al.* have suggested that in these chimeras *D. discoideum* employs two strategies to increase their fitness in social conflicts [15]. First, strains exhibit fixed strategies, which are independent on the identity of the other genotype in the aggregate. Second, facultative strategies have also been observed in which strains exhibit partner specific changes in behavior leading some to actively promote themselves to become spores or coerce the other genotype to form stalk in chimera [15]. Little is known, however, about the genetic and molecular basis of self/non-self recognition during such chimeric development, including the downstream signaling cascades, gene regulatory interactions, cellular responses and behavioral changes in chimeras and how these affect the process of social evolution. Given the possibility of kin discrimination and dominance in social interactions, a key hypothesis is that there should be differential expression of genes in *D. discoideum* individuals if they were interacting with clone mates vs. if they were in chimeras. Such changes in gene expression may serve as the transcriptional response associated with self/non-self recognition and possibly lead to kin discrimination and social dominance.

In this study, we examined genome-wide gene expression during fruiting body development of natural *D. discoideum* strains, and identified differentially expressed genes in clones vs. chimeras. We studied natural strains that were isolated from North Carolina, whose social phenotypes were well characterized and had been shown to form a dominance hierarchy when co-developing in chimeras [14, 15]. The transcriptomic profiles of *D. discoideum* cells in clone vs. different chimeras at five different developmental stages were analyzed using a customized microarray we developed. We were able to study the effects of chimerism on global transcriptional variation, and identify developmental stage-dependent gene expression patterns that were characteristic of social interactions of *D. discoideum* cells in chimeric fruiting bodies.

## Results

### Gene expression differences in D. discoideum

A customized gene expression microarray was specifically designed for *D. discoideum* via the Agilent platform, which contained 32,199 oligonucleotide probes covering 10,858 genes. These genes represents ~84% of total number of predicted genes in the genome of this species. Test RNA samples from multiple stages of NC105.1-RFP clonal development were used to validate this *D. discoideum* microarray. Technical replicates of test RNA samples showed high correlation (r > 0.93), and dye-swap experiments revealed minimal dye bias between Cy3 and Cy5 labeled samples (r < -0.91, Additional file 1: Figure S4). In addition, gene expression patterns from 11 developmentally-regulated genes detected on the *D. discoideum* microarray were confirmed by qRT-PCR (Additional file 1: Figure S1). Accumulated studies have suggested that the performance of this technology is consistent across multiple systems [16, 17]. These evidences support that this customized microarray provides a reliable and efficient tool for high-throughput gene expression analysis in *D. discoideum*.

We examined the patterns of genome-wide gene expression in *D. discoideum* cells undergoing social interactions during development upon starvation. More specifically, we examined how global gene expression patterns differed in cells that were co-developing with other cells of identical genotypes (clonal development) vs. other cells that were genotypically distinct (chimeric development). We examined four wild strains of *D. discoideum* that had been characterized in terms of social behavior in chimeras [14, 15]. The strain NC105.1 was shown to have the highest ability to promote itself in chimeric fruiting bodies to become spore and to coerce others to become stalk cells. NC85.2 was lowest in the dominance hierarchy in social competition, while NC28.1 and NC63.2 ranked in the middle of the hierarchy. To make the comparison, mixing experiments were performed between cells of the focal strain, a wild *D. discoideum* isolate from North Carolina, which had been transformed to allow for constitutive RFP expression (NC 105.1-RFP). This focal strain was studied when it underwent fruiting body development either clonally with its non-RFP parental wild strain (NC105.1) or when mixed as a chimera with one of three other wild North Carolina strains (NC28.1, NC63.2 and NC85.2) in equal ratios. Cells were collected and mechanically disassociated at five different developmental stages: the aggregate (~4 h), mound (~8 h), finger (~12 h), slug (~16 h), and culminant (~20 h) stages (Additional file 1: Figure S2). NC 105.1-RFP cells were then separated from the co-developing non-RFP cells by fluorescence-activated cell sorting (FACS), and gene expression was assayed using the Agilent microarray.

In all three sets of chimera experiments (NC105.1-RFP vs. NC28.1, vs. NC63.2, and vs. NC85.2), gene expression showed good correlation between biological replicates (0.77 < r < 0.99, mean r = 0.94). Out of the 10,858 genes present on the microarray, between ~7,000 and ~8,000 genes were detected across all three chimeric sets. The NC105.1-RFP vs. NC85.2 set had been conducted separately in time from the other two chimeric sets, and an analysis using Pearson’s correlation coefficients between three control pairs at each time point (NC105.1-RFP vs. NC105.1) revealed that the gene expression in the control pair associated with the NC85.2 experiment did not correlate well with the gene expression in the control pairs associated the other two chimeric set experiments. In contrast, control pairs of the NC105.1-RFP vs. NC28.1 set and the NC105.1-RFP vs. NC63.2 set showed good correlation in gene expression with each other at every time point (0.84 < r < 0.98). The NC105.1-RFP vs. NC85.2 set was therefore excluded from subsequent analyses to avoid the time block effect.

An ANOVA model for gene expression was specified in which measured level of gene expression was determined by mixing status (C) [e.g., whether NC105.1-RFP developed chimerically with another wild strain or clonally with the NC105.1 wild genotype], which specific strain was mixed with NC105.1-RFP (M), and developmental stage of the sample (T). The results of the ANOVA analyses are in Table 1. We find that 1,759 genes were significantly different between chimera and clone, 1,144 genes showed significant strain differences, and 6,586 genes were developmentally regulated over time (FDR q-value < 0.01). Based on the numbers of significant genes identified by each factor in the ANOVA model, our analysis suggests that developmental stage is a major source of variation in gene expression, responsible for the differential expression of the largest number of genes.

**Table 1:** **Number of statistically significant genes identified in the ANOVA analyses**

Term	Biological meaning	Number of statistically significant genes
C	Chimerism	1759
M[C]	Mix	1144
T	Time	6586
C × T	Interaction	6584
M[C] × T	Interaction	6593
C × M[C] × T	Interaction	6593

**Figure 1:**
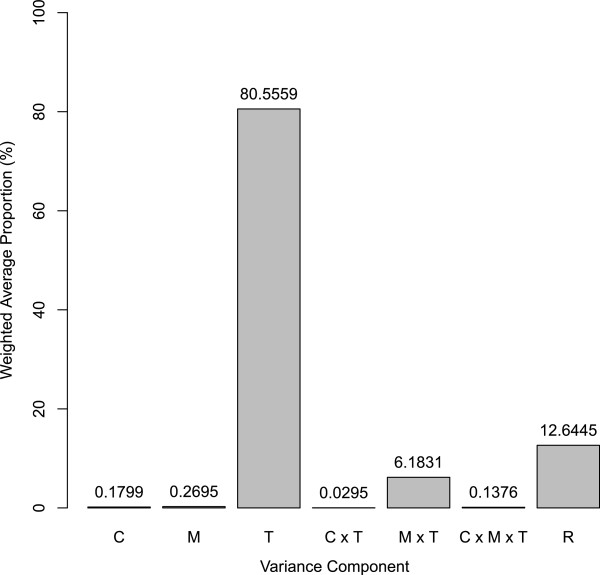
**Proportion of the transcriptional variance explained by each variance component.** C: chimerism; M: mix; T: time.

To further investigate the proportion of variation in gene expression explained by each factor, a principal variance components analysis (PVCA) was run on the same data set [18]. This approach first reduces data dimensionality with principal component analysis (PCA), and then fits a mixed linear model to each principal component with variance components analysis (VCA). The variance components are averaged across all of the principal components using the corresponding eigenvalues as weights, and the magnitude of each source of variation is presented as a proportion of total variance. Principal component 1 (PC1) and PC2 explained 45.8% and 27.4% of the variation in global gene expression, respectively. The variance component T (developmental stage/time) was the major source of variation in PC1 and PC2, and also explained a large proportion (weighted average proportion of 80.56%) of total transcriptional variance (Figure 1). This result confirmed the conclusion derived from the ANOVA analysis that developmental regulation played a significant role in modulating global gene expression. The linear model of the principal components attributed a very small amount of the transcriptional variance to chimerism-related factors (C: 0.18%, C × T: 0.03%), which suggested that the overall transcriptional difference between chimera and clone was relatively small.

**Figure 2:**
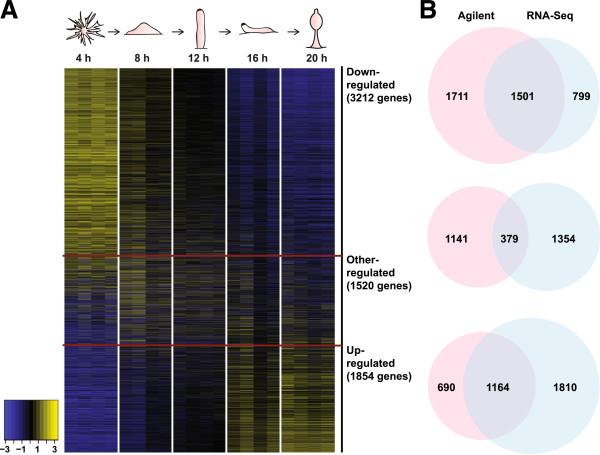
**Developmental regulation of gene expression. (A)** A transcriptional heatmap of standardized gene expression patterns of the 6,586 developmentally regulated genes, ordered according to their regulatory patterns. The colors represent relative gene expression level (see scale). Each row represents a gene and each large column represents a time point, with a figure of corresponding developmental stage on the top. Within the large column, the four small columns represent data from clonal, chimeric, clonal, and chimeric pairs, respectively. The dark red lines divide the genes into three groups: down-regulated, other-regulated, and up-regulated genes. Number of genes in each group is indicated in parenthesis. **(B)** Venn diagrams compares the numbers of genes that fall into the specific regulatory patterns identified in our study and in a previous RNA sequencing (RNA-Seq) transcriptome study (Parikh *et al.*[19]). The pink circle represents genes identified by our customized Agilent microarray, and the blue circle represents results from the RNA-Seq study. From top to bottom: down-regulated, other-regulated, and up-regulated genes.

One issue is whether gene expression in our experiments reflects patterns observed *in situ* in the developing fruiting body, even after we subject the cells to fluorescence-activated cell sorting. We compared our results to previous studies on the comparative developmental transcriptome in *D. discoideum* and *D. purpureum* using RNA sequencing (RNA-Seq) [19]. In our study, we find that 6,586 genes in the *D. discoideum* genome were developmentally regulated over time (FDR q-value < 0.01), and together with the PVCA analysis suggests that developmental stage is a major source of variation in gene expression and is responsible for the differential expression of a large number of genes. In the previous study, the abundance of almost every transcript was observed to change at least two-fold during development, similar to what we observed using our *D. discoideum* microarray. We compared three distinct developmental expression patterns in our data vs. the RNA-Seq study, and find good overlap between our study and the RNA-Seq study in up- and down-regulated genes (Figure 2).

Gene ontology (GO) enrichment analysis also showed that similar categories of genes were enriched in our microarray study in the up- and down-regulated gene clusters (Additional file 1: Table S1), as compared to the previous RNA-Seq study, which observed down-regulation of translation and cytoskeleton organization genes, as well as up-regulation of differentiation and spore development genes. In the down-regulated genes, ten GO categories that belong to biological process ontology are overrepresented (p < 0.01), which included genes associated with ubiquitin-dependent protein catabolic processes, translation, tricarboxylic acid cycle, and mRNA metabolism. These genes suggest a trend towards a metabolic decline during fruiting body development, consistent with the reaction of *D. discoideum* to starvation. Among the up-regulated gene clusters, 10 GO categories are overrepresented and include genes associated with cation transport, steroid metabolic process, protein phosphorylation, peptidyl-histidine phosphorylation, and genes involved in culmination during sorocarp development.

The overlap in genes identified in the previous RNA-Seq study and our microarray analysis is good but not perfectly congruent. This may be due to differences in time series sampling as well as specific strains used. Nevertheless, the strong overlap in our experiments as well as the results of the GO analysis suggests that the gene expression patterns we observe in our experiments reflect to a large extent the gene expression in the developing fruiting body, even after mechanical disaggregation and cell sorting.

### Effects of chimerism on gene expression in D. discoideum

**Figure 3:**
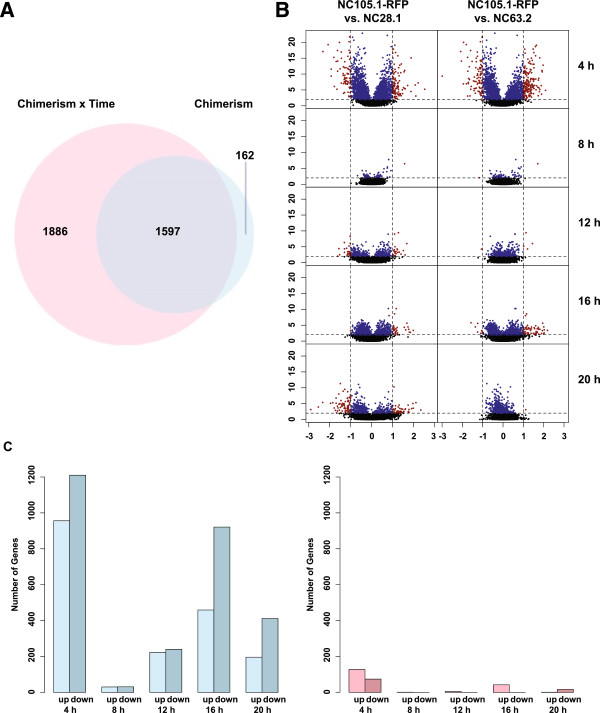
**Effects of chimerism on gene expression. (A)** Venn diagram compares significant genes identified in the ANOVA model term chimerism × time and in the term chimerism. The pink circle represents chimerism × time (C × T), and the blue circle represents chimerism **(C)**. **(B)** Gene expression volcano plots show both statistical significance and magnitude of gene expression change obtained from each experiment set and developmental time point. Each row represents a developmental stage and each column represents an experiment set. The x-axis represents the difference of M in chimera vs. in clone, where M = log_2_(Cy3/Cy5 signal intensity), and y-axis represents the log_10_(p) from the ANOVA model. The blue dots represent genes with statistically significant expression differences in clones vs. chimeras (p < 0.01) with a small differences in expression (fold-change < = 2), the red dots represent statistically significant genes (p < 0.01) with a large change in expression (fold-change > 2), and black dots represent statistically non-significant genes (p > =0.01). **(C)** Plots show numbers of significant genes identified at different developmental stages under different criteria. Left: number of genes that are statistically significant (p < 0.01). Right: number of genes that are statistically significant and with a large change in gene expression (p < 0.01 and fold-change > 2).

Chimera-associated differential genes, which show differential expression when a *D. discoideum* strain is interacting with a dissimilar genotype, were identified in the ANOVA analysis both as a main effect of chimerism (C) and an interaction effect with developmental stage (C × T). We find that 1,759 genes were differentially expressed under chimeric vs. clonal conditions, while 3,483 genes showed a significant interaction effect (C × T). Most of the genes that were significantly different in expression between chimera vs. clone were also significant in the interaction term, suggesting that stage-specific regulation was predominant during chimeric development (Figure 3). There are 3,645 genes identified by either main effect or interaction effect, and 162 genes are specifically regulated under chimeric development, with a characteristic expression pattern that features continuous small differences between chimera vs. clone over development (Additional file 1: Figure S3).

**Figure 4:**
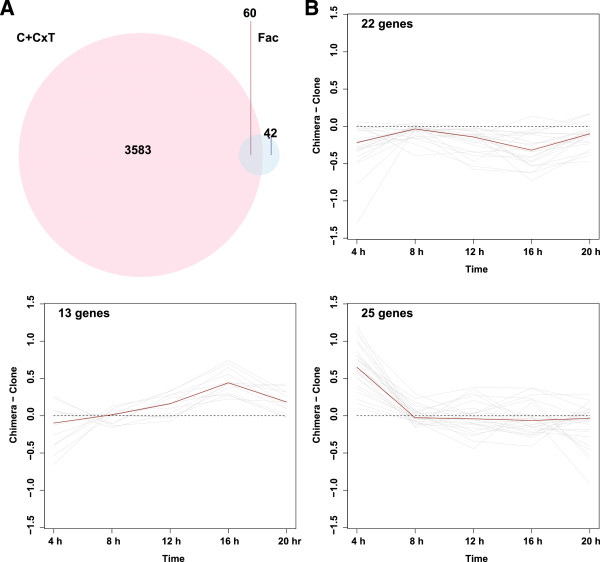
**Comparison between chimera-associated differential genes and facultative cheater genes. (A)** Venn diagram compares the chimera-associated differential genes identified on the *D. discoideum* microarray and the facultative cheater mutants revealed by previous mutant screen studies [20]. The pink circle represents genes significant in the C and C × T terms of the ANOVA model, and the blue circle represents previously-identified facultative cheater genes. **(B)** PAM cluster analysis on the 60 genes that overlap between our study and previous mutant screen studies. Expression of these genes cluster into three groups. X-axis represents time, and y-axis represents the difference of M in chimera vs. in clone, where M = log_2_(Cy3/Cy5 signal intensity). Grey lines in each panel represent trajectories of expression of every gene in the cluster, and dark red lines represent the median gene expression trajectory of the cluster. The numbers of genes in each cluster are listed in the upper left corner of each panel.

A previous genome-wide mutagenesis study identified 129 facultative cheater mutants that are overrepresented in the sorus during chimeric development while cooperating normally with other cells during clonal development [20]. One hundred and two of these genes also displayed expression in our *D. discoideum* microarray assay, and we asked how many of these facultative cheater genes were differentially expressed in chimera vs. clone. Chimerism significantly affected the expression of 60 of these previously identified facultative cheater genes, which was ~60% of the total expressed number of facultative cheater genes (Figure 4).

Cluster analysis revealed three distinct regulatory patterns among these 60 differentially expressed loci associated with facultative cheater mutants (Figure 4). Genes in cluster 1 were constantly down-regulated in chimera and the magnitude of down-regulation was maximum at the 4 h aggregate stage and the 16 h slug stage. In cluster 2, major transcriptional up-regulation in chimera was observed at the slug stage, while a peak in expression in the chimera was observed in the aggregate stage in cluster 3. These patterns indicate that facultative cheater genes undergo different modes of regulation during chimeric development, and also reveals that cheating-related chimeric regulation occurs at several stages of development.

A substantial number of genes showed significant interaction effect in expression level between chimera/clone and developmental stage. Indeed, the numbers of differentially regulated genes between chimera vs. clone vary significantly across time, with 2,166 significant loci at 4 h, 61 at 8 h, 463 at 12 h, 1,380 at 16 h, and 608 at 20 h (Figure 3). These results suggest that the 4 h aggregate stage represents not only the major developmental transition from single-cell to multicellular phase, but also where cells may potentially respond to genetic similarities/dissimilarities between each other and can differentially regulate a putative chimera recognition program. The relatively large number of genes identified at 16 h, the early onset of sporulation stage, suggests the other developmental stage that may be important for chimeric development. It must be noted, however, that most of the significant genes showed only a small change in absolute expression level, with most showing <2-fold expression differences between chimera vs. clone (Figure 3). These observations suggested that chimerism results in a complex, but possibly subtle, alteration in the developmental program involving multiple stages of differentiation and numerous genes with small effects.

### Genes associated with chimeric development

**Figure 5:**
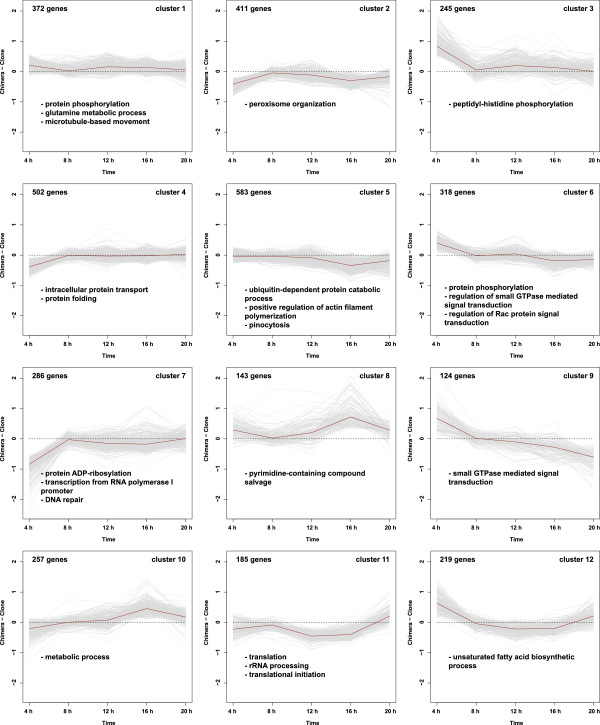
**PAM cluster analysis on the 3,645 chimera-associated differential genes, which are clustered into 12 groups.** X-axis represents time, and y-axis represents the difference of M in chimera vs. in clone, where M = log_2_(Cy3/Cy5 signal intensity). Grey lines in each panel represent expression trajectories of every gene in the cluster, and dark red lines represent the median expression trajectory of the cluster. The numbers of genes in each cluster are listed in the upper left corner of each panel. Overrepresented gene ontology (GO) categories for biological process are listed at the bottom of each panel.

Cluster analysis also suggests the complexity and diversity of chimeric regulation in *D. discoideum*. The 3,645 chimera-associated differential genes (C + C × T) identified in our analyses fall into 12 co-expressed gene clusters identified by PAM. The co-expressed gene clusters identified by PAM are associated with distinct expression patterns as well as over-representation of different functional GO categories (Figure 5). For instance, genes involving small GTPase-mediated signal transduction in cluster 9 are up-regulated in chimeras at the aggregate stage, and are gradually down-regulated in chimeras at later stages of development. Another example comes from the expression patterns of translation-related genes, which were enriched in cluster 11. The expression of genes in this cluster was suppressed in chimeras from the very early stages of development, but is up-regulated in chimeras at the late 20 h culminant stage.

Since the early aggregate stage is critical for multicellular development and apparently for differential gene regulation during chimeric development, we examined how candidate genes important in this morphological transition are regulated in chimera vs. clone. The origin of multicellularity is closely associated with cell-cell adhesion molecules (CAMs), and we analyzed the expression of this group of genes in detail [21]. There are four adhesion systems in *D. discoideum*, three of which are well-characterized [22]. EDTA-sensitive, Ca^2+^-dependent adhesion sites are mediated by the protein DdCAD-1, which is encoded by the gene *cadA*, while the two EDTA-insensitive systems include the gp80 protein, encoded by *csaA,* and gp150 encoded by *tgrC1* [23–26]. The importance of the latter two adhesion systems in social evolutionary analyses in *D. discoideum* has been widely recognized, as *csaA* is the first reported single green-beard gene, and *tgrC1* is known to mediate kin discrimination in this social microbe [12, 13, 27].

Previous studies have shown that the temporal expression of these proteins are strictly controlled. DdCAD-1 is expressed early at the onset of development, followed by the expression of gp80 [25, 28]. Low levels of gp150 are observed at mid-aggregation, and accumulation occurs during mound formation [29]. CAMs not only mediate cell-cell adhesion, but also regulate various biological processes across development, including aggregate size regulation, cell-type proportioning, and cell sorting [30–32].

**Figure 6:**
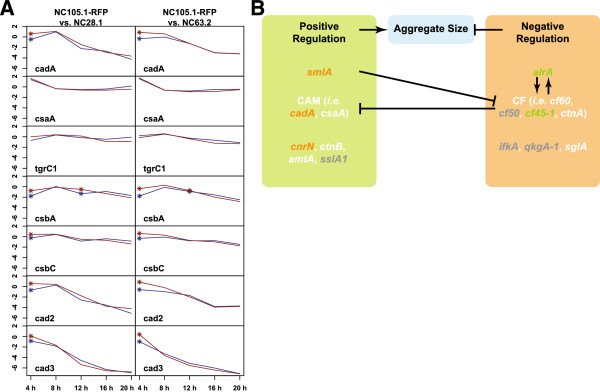
**Gene expression of CAMs and its impact on aggregate size. (A)** The gene expression patterns of cell adhesion molecules (CAMs) in chimera vs. in clone. Each row represents a gene and each column represents an experiment set. The x-axis represents time, and y-axis represents M, where M = log_2_(Cy3/Cy5 signal intensity). Red and blue lines represent gene expression patterns in chimera and clone, respectively. Asterisks represent significant differences identified by ANOVA. **(B)** Gene network that coordinately regulates aggregate size in *D. discoideum* development. Positive regulators of aggregate size are listed in the left panel, and negative regulators are on the right. Genes in orange showed up-regulation in expression level in chimera vs. in clone at the aggregate stage, while genes in green were down-regulated in expression. The expression of genes in white was not significantly different in chimera vs. clone, and the expression of genes in grey was not detected on the *D. discoideum* microarray.

The expression patterns of *cadA*, *csaA* and *tgrC1* in chimera and in clone revealed little difference. The transcription level of *cadA*, however, was significantly up-regulated at the aggregate stage (-log_10_[FDR q-value] > 4.76) (Figure 6). Expression patterns of *csbA* (-log_10_[FDR q-value] > 8.57) and *csbC* (-log_10_[FDR q-value] > 4.38), which encode components of the contact site that DdCAD-1 mediates, as well as *cad2* (-log_10_[FDR q-value] > 5.86) and *cad3* (-log_10_[FDR q-value] > 4.35), which encode putative calcium-dependent cell adhesion molecules, also showed a significant up-regulation at the aggregate stage in chimeras (Figure 6). There were 16 other genes sharing a similar expression pattern over time with *cadA* (r > 0.9 under all pairing conditions), a few of which were of unknown function (Table 2). Transcription levels of all these genes were also significantly up-regulated at 4 h in chimeras.

**Table 2:** **Genes sharing a similar expression pattern over time with** ***cadA***

Gene ID	Gene name	Gene product
DDB_G0290347	n.a.	Zinc finger RING-type pattern-containing protein
DDB_G0280413	n.a.	
DDB_G0274391	*alfA*	Alpha-L-fucosidase
DDB_G0277407	n.a.	
DDB_G0267456	*cbp2*	Calcium-binding protein
DDB_G0291081	*pefA*	Penta-EF hand calcium binding protein
DDB_G0287847	n.a.	U box domain-containing protein; putative E3 ubiquitin-protein ligase
DDB_G0279681	n.a.	Calcium-binding EF-hand domain-containing protein
DDB_G0293704	n.a	Seven transmembrane domain protein
DDB_G0276965	*gltA*	Citrate synthase
DDB_G0290527	n.a.	
DDB_G0283091	n.a.	FNIP repeat-containing protein
DDB_G0289549	n.a.	
DDB_G0280865	n.a.	
DDB_G0293522	*ponA*	Actin binding protein; ponticulin
DDB_G0283089	n.a.	
DDB_G0275439	*cad2*	Putative adhesion molecule

n.a, not applicable.

These findings suggest that there might be a common regulatory mechanism governing the expression of these genes, which results in an increased level of cell-cell adhesion after the initiation of development in chimeras. CAMs are known to regulate aggregate size at early developmental stages, and increased cell-cell adhesion leads to larger aggregates [30, 33]. We examined if gene expression levels of other aggregate size regulators are coordinately altered in chimera and thus potentially affect aggregate size. DdCAD-1 is regulated by a large secreted protein complex called counting factor (CF), a negative regulator of aggregate size [30]. The gene *cf45-1*, which encodes a component essential for CF function, is down-regulated (-log_10_[FDR q-value] > 4.20) at the 4 h aggregate stage in chimeras. Examination of the upstream regulators of CF revealed an interesting complementary pattern: *smlA*, a negative regulator of CF, was transcriptionally up-regulated (-log_10_[FDR q-value] > 4.07) at the aggregate stage in chimeras, while *alrA*, a positive regulator of CF was down-regulated (-log_10_[FDR q-value] > 7.14) [32, 33]. The patterns of expression in this aggregate size gene network thus predicts an increase in aggregate size in chimera vs. clone as a result of up-regulation of positive size regulators and down-regulation of negative size regulators (Figure 6).

## Discussion

When the development of multicellular structures in organisms such as *D. discoideum* can include cells with dissimilar genotypes, cells of the same genetic background employ different strategies to succeed in competition. In these circumstances, a basic requirement for cells is to distinguish genetically identical and dissimilar individuals, with associated levels of cooperation over the course of the social interaction. These raise several questions concerning chimeric development in *D. discoideum*. How is the assessment of genetic relatedness achieved, and how is the appropriate response coupled with the interaction between individuals? If a cell in a *D. discoideum* fruiting body can recognize self vs. non-self interactions (in clonal vs. chimeric development), is there a chimera developmental program in social amoeba that differs from the clonal program? What are the genes that are triggered in this hypothetical chimera developmental program?

Previous studies have focused on examining the functional effect of single genes on social interactions in *D. discoideum*, using mutant lines obtained from screening in laboratory strains [20, 34, 35]. The social cycle of *D. discoideum*, however, is a complex and interactive process, where the function of a single gene may represent only one node of a social gene network. It is likely that social interactions may lead to modifications of the developmental program, which in turn arise through changes in transcriptional gene regulation. Our study dissected transcriptomic profiles of a natural strain of *D. discoideum* in clonal vs. chimeric multicellular structures during development, and found significant alteration of gene expression levels. We find, however, a relatively small proportion of over-all variation in gene expression is explained by differences between chimeric and clonal development, especially when compared to the large-scale changes in gene expression associated with the developmental process in this species. The relatively small modifications in gene expression associated with chimerism, however, is compatible with the high level of cooperation observed among different strains of *D. discoideum*; cells of distinct genetic backgrounds will co-aggregate indiscriminately and co-develop into fruiting bodies [36]. In contrast, different strains of a related Dictyostelid species, *D. purpureum*, were found to cooperate poorly in the development of fruiting bodies due to severe kin discrimination [36].

We identified the number and nature of genes involved in the putative chimera developmental program in *D. discoideum*. Interestingly, we find almost half of the transcriptome shows significant differences in gene expression between chimera and clone, though again it should be noted that the changes in absolute expression levels are small. This suggests that chimeric development may involve re-programming of the transcriptome through small modifications of the developmental genetic network, which may also indicate that response to social interaction involves many genes with individually small transcriptional effect.

One may argue that the small change in gene expression levels (albeit across a large number of genes) is due not to active recognition of non-self strains in chimeric fruiting bodies, but by a completely passive process [37]. It is difficult, however, to distinguish passive inherent response to chimeric development from active facultative response during social conflicts. Moreover, it has been noted that both active and passive recognition and response are aspects of plausible evolutionary strategies that are likely to be employed by *D. discoideum* [15].

One possible consequence of these findings is that it suggests that there may be numerous opportunities for cheater genotypes to arise and invade a population, given the large number of genes that are differentially regulated during chimera development. These potential cheaters may arise both from mutations of several genes in the same regulatory network and from single mutations that result in large effects on global development. Indeed, it has been demonstrated that cheater strains might not be rare in nature [20], which is consistent with the possibility that more cheaters may be identified by intensive genetic screens [38, 39].

We also observe a strong temporal pattern in gene expression differences during chimeric development. Among genes associated with chimeric development, nearly half were differentially expressed at specific developmental stages instead of across the entire developmental time course. Specifically, almost 60% of the chimera-associated differential genes were differentially expressed at the 4 h aggregate stage, which corresponds to the initial transition of *D. discoideum* from solitary life to a multicellular phase. At this early developmental stage, direct cell contacts begin to form, which potentially could be the first steps in self-recognition as well as kin discrimination, and thus trigger other cellular responses at later stages.

Specifically, we found that changes in gene expression at the 4 h aggregate stage in chimeras may be associated with larger aggregate size in *D. discoideum*. This finding is consistent with phenotypic observations that larger aggregates are favored under chimeric condition. Previous studies have suggested that chimeric slugs may be larger than clonal slugs in nature because more swarming cells are available [40]. It has been pointed out that these larger chimeric slugs could be favored by natural selection, since several possible ecological benefits accrue from increased aggregate size [6, 9]. Larger slugs are able to travel further and increase the chance for chimeras to culminate at a location better suited for dispersal. Moreover, larger fruiting bodies contain more spores, which increases the absolute number of cells that can be dispersed. Finally, it has been suggested that chimeras tend to produce a shorter stalk compared to clones of the same size [15], and larger fruiting bodies compensate for this by having longer stalks, which are considered to protect spores from hazards in the soil and to facilitate the dispersal of spores by lifting them above ground.

Our findings provide the first overview on the effects of chimerism on global gene expression during the development of *D. discoideum*. The mechanism by which chimerism results in gene expression differences remains to be elucidated, and one can imagine two modes. One mode is an active recognition of dissimilar genotypes by interacting cells that results in modified developmental behavior. Another mode is a more passive mechanism, by which the occurrence of specific cell-cell interactions (e.g., cell adhesion) is genotype specific, but not necessarily part of an evolved recognition system. In a sense, both these alternate modes still represent responses to chimeric vs. clonal interactions, but can have different consequences for subsequent development. More work is necessary to examine more closely whether the response to chimerism is part of an active recognition system.

Our study is further limited in examining changes in the genome-wide gene expression of only one strain interacting either with itself or two other wild strains. Expanding the analysis to other strain combinations could reveal genotype-phenotype correlations, and further distinguish generalized chimera-associated transcriptional responses from other strain-specific changes in gene expression. It would also be of interest to examine the molecular changes that may occur in the wild, where the conditions of growth (e.g., density, contact with non-self genotypes, nutrition) are undoubtedly different. Despite these limitations, our analyses revealed a list of putative chimera developmental genes amenable for functional analysis, such as genes that share a transcriptional regulatory pattern with the cell-cell adhesion gene *cadA*. A large proportion of these genes are of unknown function, and detailed examination may reveal their role in mediating social interactions. Indeed, through a combination of genomic and genetic studies, coupled with evolutionary and ecological approaches, we may identify the social gene network in *D. discoideum*, associated with these and other loci, and provide greater understanding of the biology of social cooperation and conflict.

## Conclusions

Our study is able to define the transcriptional changes associated with chimeric development of identical vs.non-identical genotypes of the social amoeba *Dictyostelium discodeum*. We find that a relatively small proportion of transcriptional variation in gene expression is explained by differences between chimeric and clonal development. The relatively small modifications in gene expression associated with chimerism is compatible with the high level of cooperation observed among different strains of *D. discoideum*; cells of distinct genetic backgrounds will co-aggregate indiscriminately and co-develop into fruiting bodies. Chimeric development may involve re-programming of the transcriptome through small modifications of the developmental genetic network, which may also indicate that response to social interaction involves many genes with individually small transcriptional effect.

## Methods

### Microarray design and validation

A total of 12,836 coding sequences (version 01/10/2011) was obtained from DictyBase (http://dictybase.org/) representing the entire transcriptome [41]. After removing all transposable elements and keeping only common regions of transcriptional variants, 12,290 sequences were used to design probes using eArray (https://earray.chem.agilent.com/earray/) for an Agilent Custom Gene Expression Microarray (Santa Clara, CA). Three oligonucleotide probes 60 nts in length were generated for each transcript. Probes with poor Base Composition (BC) Score, a numeric value that defines the quality of the probe based upon its base composition and distribution, or showing cross-hybridization, e.g., multiple Megablast hits when searched against the *D. discoideum* genome, were excluded from further consideration. In the end, a probe set representing 10,858 genes by 32,199 probes was synthesized on an Agilent microarray. Each slide consisted of four replicates of this microarray design. Each microarray was capable of harboring 44,000 probes, including customized probes and standard control probes, so some spots on the array were not used in our design. Test RNA samples were prepared from clonally developing NC105.1-RFP cells at 0 h, 4 h, 8 h, 12 h, 16 h and 20 h and hybridized against a common biological RNA reference sample derived from mixing RNA from all six time points. Two levels of technical replications were performed using these test RNA samples: one with the same sample hybridized on multiple arrays with the same dye, and the other with the same sample labeled with two different dyes. Same RNA samples were also tested with qRT-PCR on 11 developmentally regulated genes, i.e., DDB_G0279439, DDB_G0286411, DDB_G0276761, DDB_G0277141, DDB_G0278721, DDB_G0289073, DDB_G0277853, DDB_G0276887, DDB_G0271564, DDB_G0267412, DDB_G0276939, using the LightCycler^®^ 480 Real-Time PCR System from Roche Applied Science (Indianapolis, IN). Control qRT-PCR was also performed without reverse transcriptase. It was confirmed that the extent of DNA contamination was non-detectable in RNA extracts used in all experiments.

### Sample preparation and verification

Four wild *D. discoideum* isolates NC105.1, NC28.1, NC63.2, NC85.2, which were originally collected from Little Butt’s Gap, North Carolina and a transgenic strain NC105.1-RFP, which was derived from NC105.1 and constitutively expresses RFP, were grown on SM agar plates spread evenly with *Klebsiella aerogenes* (Ka). Approximately 1 × 10^5^ spores were mixed with Ka and incubated at 22°C for approximately 44 hours. For each strain, resulting amoebae were scraped off three SM agar plates, pooled together, and separated from Ka by repeated centrifugation and washing in KK2 buffer (16.1mM KH_2_PO_4_, 3.7mM K_2_HPO_4_). CellTracker™ Green CMFDA (5-Chloromethylfluorescein diacetate) from Invitrogen (Eugene, OR) was used to dye-mark non-RFP amoebae, which helped to increase contrast during cell sorting. One pooled amoebae solution was prepared for each strain. Equal amounts of RFP and non-RFP amoebae were mixed and spread evenly on two developing plates (1.5% KK2 agar) at a density of 1.6 × 10^6^ cells/cm^2^ as biological replicates. Chimeric pairs were prepared by mixing equal amount of NC105.1-RFP and the other wild strain in the experiment set, i.e. NC28.1, NC63.2, or NC85.2, while control pairs were prepared by mixing equal amount of NC105.1-RFP and NC105.1. An experiment set was composed of one of the three chimeric pairs and its corresponding control pair, and there were three experiment sets in the study.

Developing plates were incubated in a dark and humid environment at 22°C for 4 h, 8 h, 12 h, 16 h, and 20 h, respectively, before harvest. One sample was collected from one developing plate, therefore for each experiment set, i.e. a chimeric pair + corresponding control pair, there are 20 independent samples in total: two chimeric replicates and two control replicates at each of the five time points. Four samples belonging to the same experiment set with same incubation time were handled at the same time in the same environment. Samples collected at these different time points/developmental stages were mechanically disaggregated in disassociation buffer (KK2, 20mM EDTA) and subject to fluorescence-activated cell sorting (FACS) with a FACS Aria from BD Biosciences (San Jose, CA) immediately with a nozzle size of 70 μm. A set of RFP-positive cells not present in control cell populations but evident in cell suspensions made from NC105.1-RFP containing cells was collected. A set of RFP-negative cells was also collected at the same time. Gates were set using RFP-positive and dye-marked RFP-negative cells on scatter plot of PE vs. FITC channels to distinguish RFP signal and green fluorescence vs. autofluorescence. A figure showing a sample separation under FACS sorting is shown in Additional file 1: Figure S5. Total RNA of RFP-positive and RFP-negative cell populations was extracted by QIAGEN RNeasy Micro Kit (Valencia, CA) and kept at -80°C. For each developing plate, it takes ~45 minutes to complete the procedure from aggregate collection to RNA extraction. All samples are kept on ice at all times during the procedure. To verify the FACS results, RFP enrichment was examined by qRT-PCR in both RFP-positive and RFP-negative cell populations using a pair of RFP-specific primers (Forward primer: 5′- GTAAAGCATATGTTAAACATCCAGC-3′; reverse primer: 5′- ACAACACCACCATCTTCAAA-3′). At least 5-fold and up to >100-fold enrichment in RFP-expression was observed in RFP-positive cell population. Total RNA of RFP-negative cell populations were only collected and used for qRT-PCR verification, but not in microarray hybridization. Experimental design of this study meant to compare how the transcriptomic profile of the focal strain (NC105.1-RFP) changes during clonal vs. chimeric development.

### Microarray hybridization and data pre-processing

A mix of RNA samples collected at 0 h, 4 h, 8 h, 12 h, 16 h, and 20 h during clonal development of NC105.1-RFP served as the common RNA reference across all microarray experiments. Positive control transcripts, i.e. spike-in transcripts complementary to the standard control probes on microarray, were added to high quality total RNA samples and common biological reference (RNA Integrity Number [RIN] > 9.6) using the Agilent RNA Spike-In Kit for monitoring the microarray workflow. After sample amplification and labeling using the Agilent Low Input Quick Amp Labeling Kit, the quantity and quality of the coding RNA (cRNA) was assessed by both NanoDrop ND-1000 spectrophotometer, which measures the extinction at 260 nm, and the Agilent 2100 Bioanalyzer with lab-on-a-chip technology. Equal amounts of sample and common biological reference cRNA were hybridized against the same microarray at 65°C for 17 hours. Dye-swap experiments were performed for each sample, i.e. technical replicates of the same sample were prepared with both Cy3 and Cy5. The assignments of samples on microarray slides are shown in Additional file 1: Table S2. Microarray slides were washed and then scanned using Agilent’s High-Resolution C Scanner.

Gene expression information was extracted from microarray scan data by Agilent Feature Extraction (FE) software, which also detected and removed spatial gradients as well as local backgrounds. Genes identified as well above background signal intensity by FE were considered detected. Gene expression data was log_2_-transformed and normalized within array using a combined rank consistency filtering with LOWESS intensity normalization in FE. Between-array normalization was implemented using the Aquantile method in the Limma package in R (http://www.R-project.org/) to ensure the A-values (average intensities) have the same empirical distribution across arrays leaving the M-values (log_2_-ratios) unchanged [42–44].

### Statistical analysis

Analysis of Variance (ANOVA) and Principal Variance Components Analysis (PVCA) analyses were implemented using the JMP Genomics 5.1 program from SAS Institute (Cary, NC) on the full data set. The following fixed effects ANOVA model was fit to gene expression:


where y = gene expression, C = chimerism (2 levels), M = mix (4 levels, nested chimerism, control pairs of each experiment set were treated as a separate level), T = developmental stage/time (5 levels).

A False Discovery Rate (FDR) q-value < 0.01 threshold was applied to identify genes that showed statistically significant differences in gene expression from comparisons of interest. The same factors were used in PVCA, which firstly reduces the dimensionality of the data set with Principal Components Analysis (PCA), and then fits a mixed linear model to each principal component to partition variability with Variance Components Analysis (VCA) [18]. A summary of variance components across all principal components is constructed as a weighted average of the individual estimates, using eigenvalues as weights.

### Cluster analysis

Cluster analyses using the Partitioning Around Medoids (PAM) algorithm were performed on the mean expression values of the developmental genes and chimera-associated differential genes identified from the ANOVA analysis, which was implemented using the cluster package in R [45, 46]. The algorithm PAM first computes k representative objects called medoids, which represent the structure of the data set. After a set of k medoids is found, each observation of the data set is assigned to the nearest medoid to construct k clusters. The goal is to find k representative objects that minimize the objective function, which is the sum of the dissimilarities of all observations to their closest medoid. To determine the number of clusters, the gap statistic, a goodness of clustering measure, is calculated for each number of clusters k. The gap statistic was also computed using the cluster package in R, which compares within-cluster dispersion with expected dispersion under a reference null distribution simulated with 100 bootstrap replicates. The optimal number of clusters k is determined by the global maximum of the gap statistics. A Gene Ontology (GO) enrichment analysis was followed to identify association with gene ontologies for each cluster using the topGO package in R [47].

### Availability of supporting data

The data set(s) supporting the results of this article is (are) available in the Gene Expression Omnibus (GEO) repository, [GEO:GSE57212 http://www.ncbi.nlm.nih.gov/geo/query/acc.cgi?acc=GSE57212].

## Electronic supplementary material

Additional file 1: **Includes supplementary figures and tables.** (PDF 2 MB)
