# Supplementary material for: Sociogenomics of self vs. non-self cooperation during development of Dictyostelium discoideum
Source: BMC Genomics. 2014 Jul 21;15(1):616. doi: 10.1186/1471-2164-15-616 (PMC4118049; doi:10.1186/1471-2164-15-616)
Supplement: Supplementary file 1 — Additional file 1: Includes supplementary figures and tables. (PDF 2 MB) [file 12864_2014_6295_MOESM1_ESM.pdf]

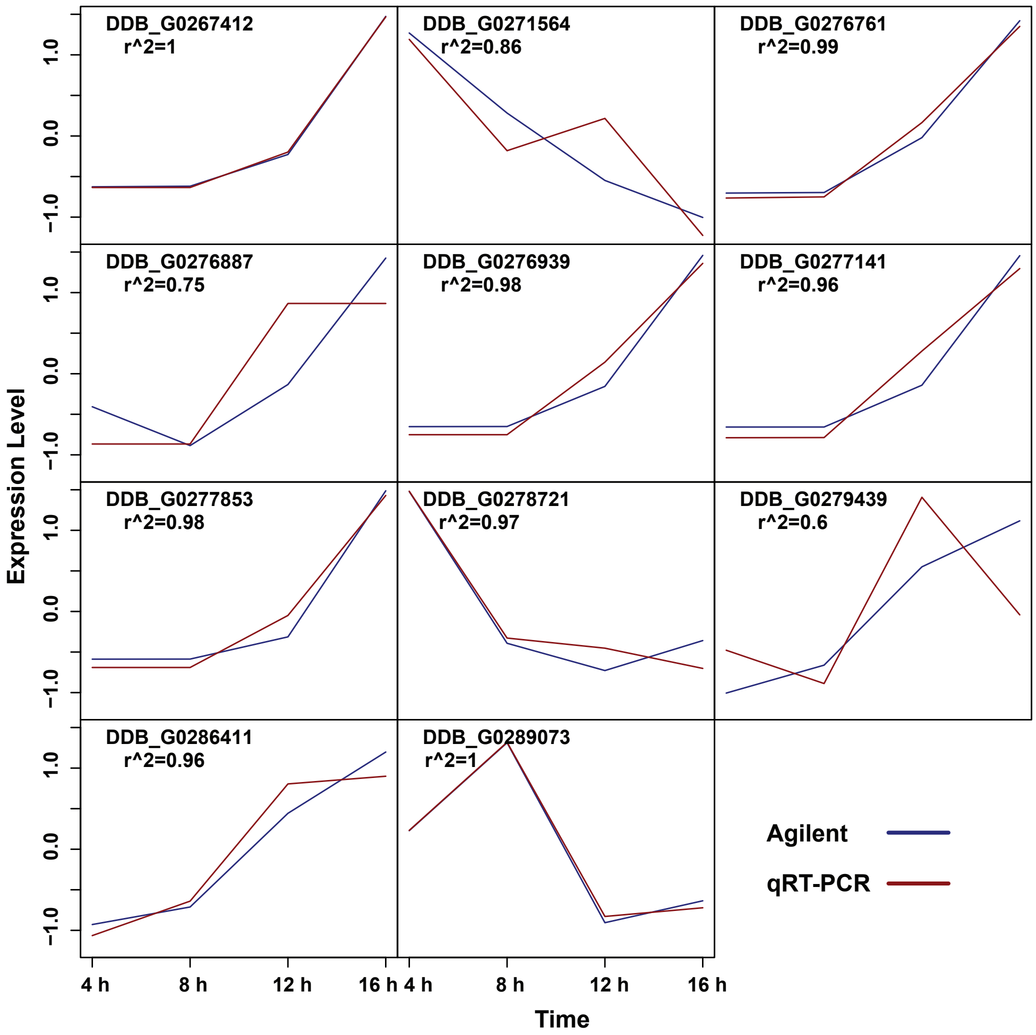

Figure S1 Comparison of individual gene expression patterns between the *D. discoideum* microarray and qRT-PCR analysis. Gene ID and Pearson's correlation coefficient between the two studies are indicated on top of each panel.

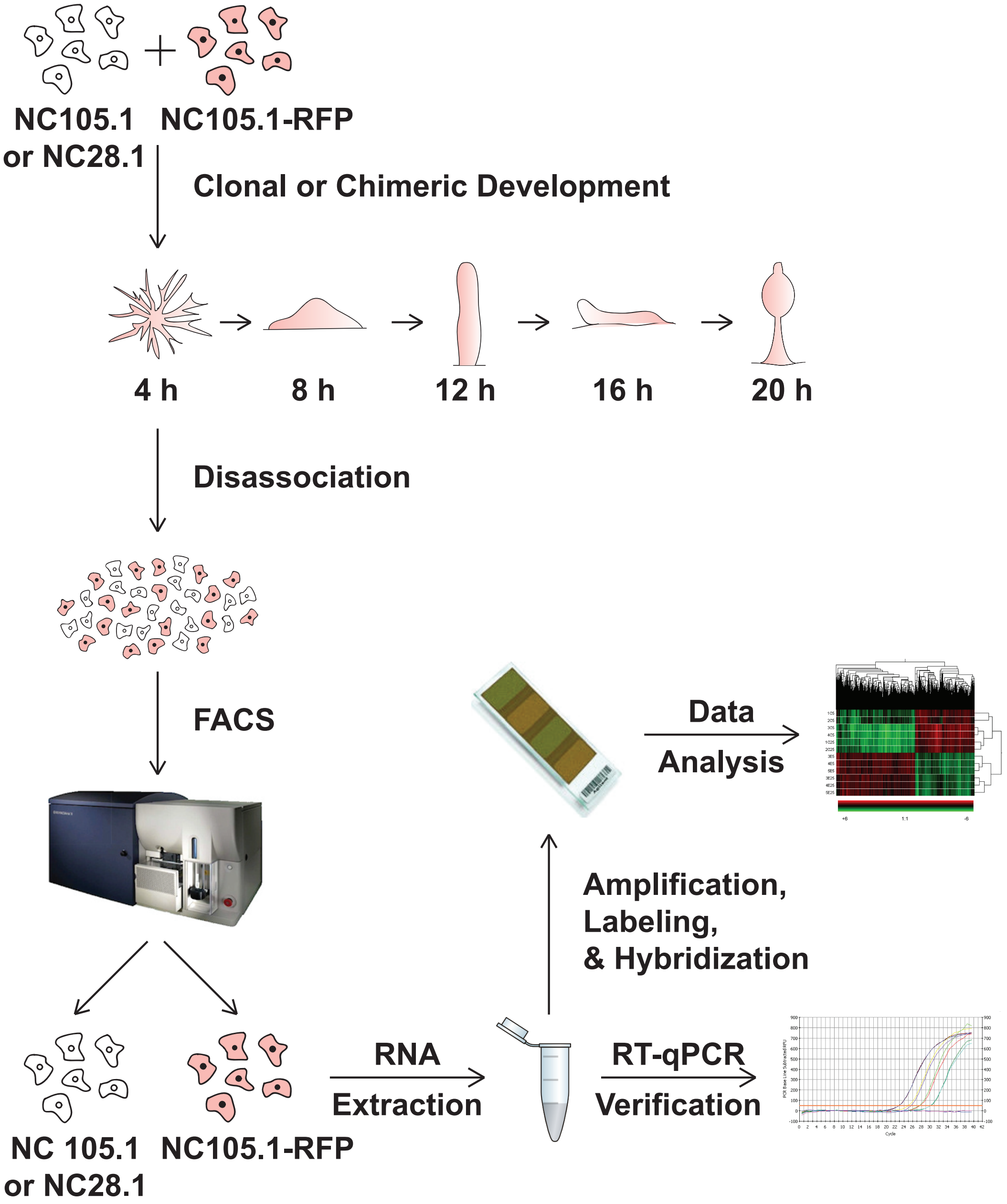

Figure S2 Experimental workflow of transcriptomic profiling during chimeric development. The example is a mixture of NC105.1-RFP and NC28.1. Equal number of cells of two genotypes were allowed to co-develop for different lengths of time and harvested at 4-hour intervals. The multicellular structures are disassociated and sorted by FACS into RFP-positive and RFP-negative populations. Once the identity of the sorted cell population is verified by qRT-PCR, the RNA samples of NC105.1-RFP are hybridized to the *D. discoideum* microarray.

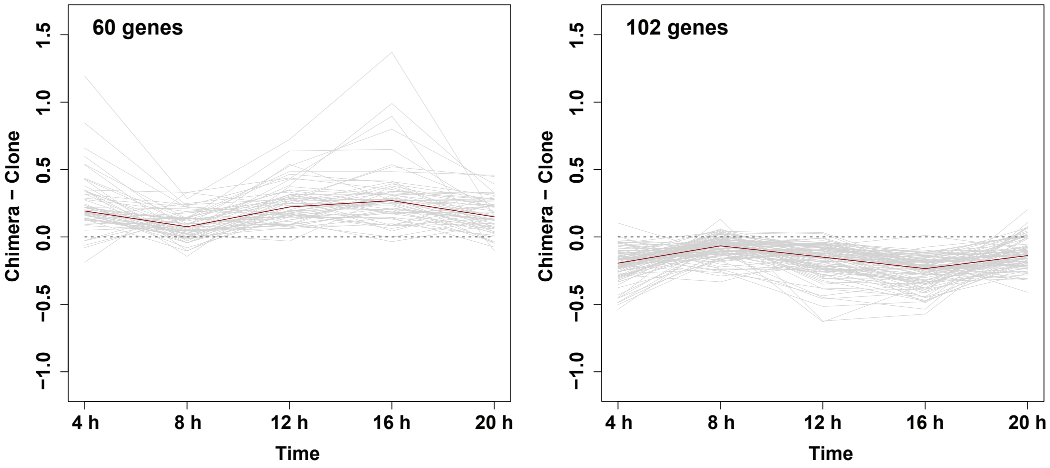

Figure S3 PAM cluster analysis on the chimeric genes identified only in the ANOVA model term chimerism. The 162 unique genes are clustered into two groups. X-axis represents time, and y-axis represents the difference of M in chimera vs. in clone, where M = $\log_2$(Cy3/Cy5 signal intensity). Grey lines in each panel represent expression trajectories of every gene in the cluster, and dark red lines represent the median expression trajectory of the cluster. The numbers of genes in each cluster are listed in the upper left corner of each panel.

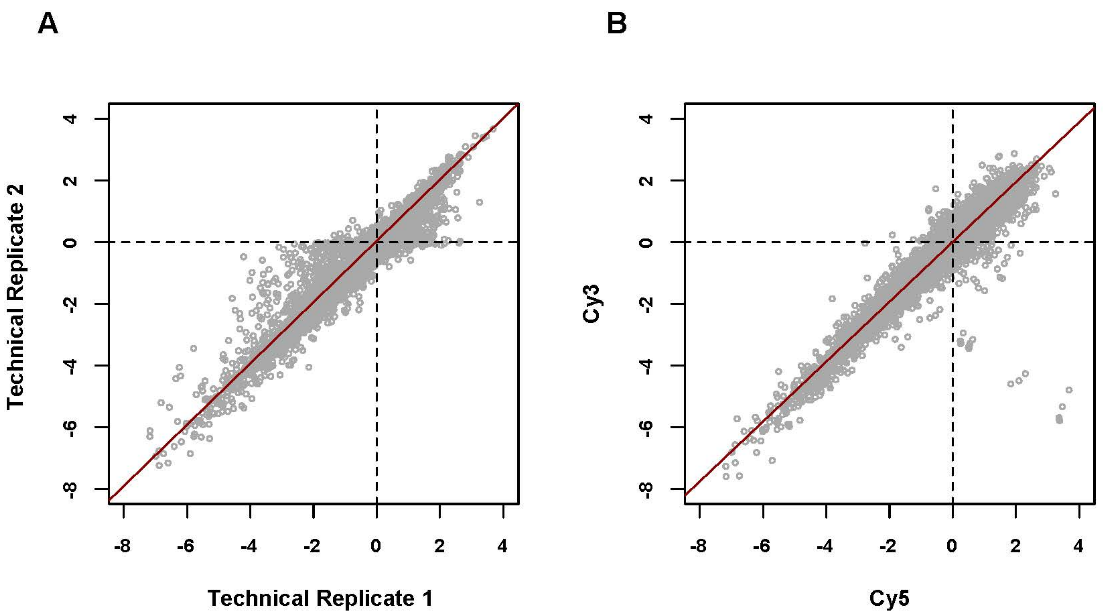

Figure S4 Correlation plots of signal intensities between (A) technical replicates and (B) dye-swaps. Axes labeled with Technical Replicate 1, Technical Replicate 2, and Cy5 represent $M = \log_2$(Cy3/Cy5 signal intensity), where as the Cy3 axis represents $-M = \log_2$(Cy5/Cy3 signal intensity). Grey dots in each panel represent genes detected on the array, and dark red lines represent the regression lines of the two variables in each panel.

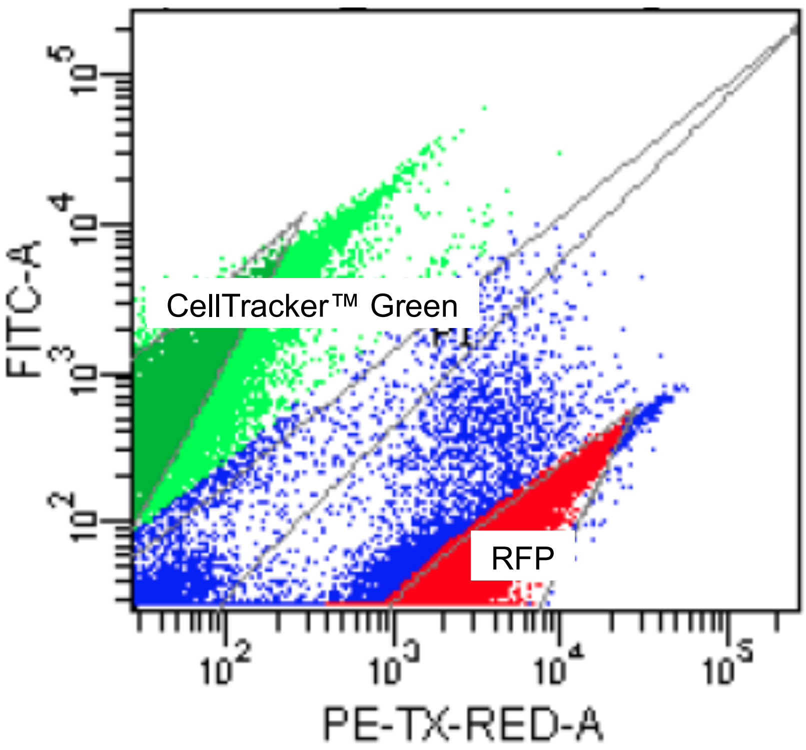

Figure S5. Gates used to separate RFP-positive cells from dye-marked RFP-negative cells.

Table S1 Overrepresented GO categories of the developmentally regulated genes

Cluster	Domain	GO.ID	Term	Annotated/Significant /Expected	Fisher.weight01
1	BP	GO:0006511	ubiquitin-dependent protein catabolic pr...	64/50/21.27	8.50E-12
1	BP	GO:0006412	translation	184/86/61.15	2.90E-05
1	BP	GO:0006099	tricarboxylic acid cycle	17/14/5.65	4.30E-05
1	BP	GO:0030163	protein catabolic process	76/60/25.26	0.00043
1	BP	GO:0016071	mRNA metabolic process	94/46/31.24	0.00127
1	BP	GO:0045454	cell redox homeostasis	20/13/6.65	0.00354
1	BP	GO:0045010	actin nucleation	15/11/4.99	0.00387
1	BP	GO:0006122	mitochondrial electron transport, ubiqui...	5/5/1.66	0.00404
1	BP	GO:0000398	nuclear mRNA splicing, via spliceosome	40/23/13.29	0.00587
1	BP	GO:0051726	regulation of cell cycle	39/21/12.96	0.00972
1	MF	GO:0004175	endopeptidase activity	70/40/23.22	5.10E-07
1	MF	GO:0003735	structural constituent of ribosome	100/55/33.17	4.60E-06
1	MF	GO:0009055	electron carrier activity	24/16/7.96	0.00078
1	MF	GO:0008137	NADH dehydrogenase (ubiquinone) activity	10/8/3.32	0.00325
1	MF	GO:0008121	ubiquinol-cytochrome-c reductase activit...	5/5/1.66	0.004
1	MF	GO:0004221	ubiquitin thiolesterase activity	21/13/6.97	0.00639
1	MF	GO:0004579	dolichyl-diphosphooligosaccharide-protei...	7/6/2.32	0.00663
1	CC	GO:0005839	proteasome core complex	16/15/5.36	2.20E-06
1	CC	GO:0005829	cytosol	141/73/47.22	2.30E-05
1	CC	GO:0005838	proteasome regulatory particle	9/9/3.01	5.20E-05
1	CC	GO:0045335	phagocytic vesicle	247/111/82.72	7.30E-05
1	CC	GO:0008180	signalosome	8/8/2.68	0.00016
1	CC	GO:0005840	ribosome	86/48/28.8	0.00017
1	CC	GO:0005885	Arp2/3 protein complex	9/8/3.01	0.00099
1	CC	GO:0005832	chaperonin-containing T-complex	8/7/2.68	0.00265
1	CC	GO:0046540	U4/U6 x U5 tri-snRNP complex	12/9/4.02	0.00395
1	CC	GO:0005643	nuclear pore	16/11/5.36	0.00414
1	CC	GO:0030126	COPI vesicle coat	5/5/1.67	0.00419
1	CC	GO:0005688	U6 snRNP	7/6/2.34	0.00699
1	CC	GO:0008250	oligosaccharyltransferase complex	7/6/2.34	0.00699
1	CC	GO:0015935	small ribosomal subunit	10/8/3.35	0.00843
1	CC	GO:0000151	ubiquitin ligase complex	24/13/8.04	0.00901
2	BP	GO:0051056	regulation of small GTPase mediated sign...	140/55/33.21	0.0014
2	BP	GO:0006935	chemotaxis	72/28/17.08	0.0023
2	BP	GO:0042593	glucose homeostasis	4/4/0.95	0.0032

2	BP	GO:0035020	regulation of Rac protein signal transdu...	42/18/9.96	0.0046
2	BP	GO:0048015	phosphatidylinositol-mediated signaling	9/6/2.13	0.0075
2	MF	GO:0005544	calcium-dependent phospholipid binding	5/5/1.17	0.0007
2	MF	GO:0005085	guanyl-nucleotide exchange factor activi...	78/32/18.25	0.00085
2	MF	GO:0030675	Rac GTPase activator activity	38/18/8.89	0.00099
2	MF	GO:0003735	structural constituent of ribosome	100/36/23.4	0.00276
2	MF	GO:0003779	actin binding	85/29/19.89	0.00503
2	MF	GO:0016303	1-phosphatidylinositol-3-kinase activity	7/5/1.64	0.0095
2	MF	GO:0016491	oxidoreductase activity	289/84/67.62	0.00974
2	CC	GO:0005615	extracellular space	243/92/57.69	2.50E-07
2	CC	GO:0016592	mediator complex	19/10/4.51	0.0059
2	CC	GO:0005886	plasma membrane	83/29/19.7	0.0096
3	BP	GO:0006812	cation transport	89/32/22.68	0.00012
3	BP	GO:0008202	steroid metabolic process	17/10/4.33	0.00043
3	BP	GO:0006468	protein phosphorylation	229/88/58.36	0.00087
3	BP	GO:0018106	peptidyl-histidine phosphorylation	13/9/3.31	0.00113
3	BP	GO:0031154	culmination involved in sorocarp develop...	51/27/13	0.00121
3	BP	GO:0045595	regulation of cell differentiation	14/8/3.57	0.0015
3	BP	GO:0030435	sporulation resulting in formation of a ...	43/22/10.96	0.00272
3	BP	GO:0030198	extracellular matrix organization	4/4/1.02	0.0042
3	BP	GO:0031149	sorocarp stalk cell differentiation	13/7/3.31	0.00466
3	BP	GO:0006355	regulation of transcription, DNA-depende...	154/47/39.24	0.00609
3	MF	GO:0031012	extracellular matrix	4/4/1.01	0.004
3	MF	GO:0016021	integral to membrane	522/155/131.34	0.0043
3	CC	GO:0015662	ATPase activity, coupled to transmembran...	21/14/5.39	0.00095
3	CC	GO:0030248	cellulose binding	5/5/1.28	0.0011
3	CC	GO:0000156	two-component response regulator activit...	18/11/4.62	0.00153
3	CC	GO:0003700	sequence-specific DNA binding transcript...	74/31/18.98	0.00154
3	CC	GO:0005275	amine transmembrane transporter activity	7/5/1.8	0.00431
3	CC	GO:0005201	extracellular matrix structural constitu...	4/4/1.03	0.00431
3	CC	GO:0043565	sequence-specific DNA binding	60/25/15.39	0.00464
4	BP	GO:0006986	response to unfolded protein	17/10/2.83	9.60E-05
4	BP	GO:0006457	protein folding	90/29/14.97	0.00018
4	BP	GO:0006260	DNA replication	31/16/5.16	0.00031
4	BP	GO:0016070	RNA metabolic process	394/119/65.52	0.00054
4	BP	GO:0006272	leading strand elongation	6/5/1	0.00065
4	BP	GO:0000375	RNA splicing, via transesterification re...	44/15/7.32	0.00074

4	BP	GO:0006364	rRNA processing	11/8/1.83	0.00193
4	BP	GO:0006334	nucleosome assembly	13/7/2.16	0.00232
4	BP	GO:0016573	histone acetylation	5/4/0.83	0.00329
4	BP	GO:0006360	transcription from RNA polymerase I prom...	8/5/1.33	0.00452
4	BP	GO:0006432	phenylalanyl-tRNA aminoacylation	3/3/0.5	0.00458
4	BP	GO:0030488	tRNA methylation	3/3/0.5	0.00458
4	BP	GO:0006400	tRNA modification	11/9/1.83	0.00839
4	MF	GO:0003676	nucleic acid binding	576/172/96.94	1.90E-07
4	MF	GO:0003677	DNA binding	227/62/38.2	8.20E-05
4	MF	GO:0008026	ATP-dependent helicase activity	38/19/6.4	0.00059
4	MF	GO:0003743	translation initiation factor activity	39/15/6.56	0.00099
4	MF	GO:0003887	DNA-directed DNA polymerase activity	10/6/1.68	0.00254
4	MF	GO:0004402	histone acetyltransferase activity	5/4/0.84	0.00345
4	MF	GO:0005524	ATP binding	532/112/89.54	0.004
4	MF	GO:0004826	phenylalanine-tRNA ligase activity	3/3/0.5	0.00475
4	MF	GO:0008270	zinc ion binding	378/82/63.62	0.00614
4	MF	GO:0051082	unfolded protein binding	46/15/7.74	0.00638
4	MF	GO:0003924	GTPase activity	43/14/7.24	0.00842
4	CC	GO:0005730	nucleolus	28/19/4.68	1.60E-06
4	CC	GO:0005634	nucleus	593/157/99.22	0.00017
4	CC	GO:0005736	DNA-directed RNA polymerase I complex	7/5/1.17	0.00202
4	CC	GO:0008023	transcription elongation factor complex	8/6/1.34	0.00869

Table S2   Sample assignments on microarray slides

	Slide 1	Slide 2	Slide 3	Slide 4	Slide 5
**Array 1**					
Experiment Set	NC105.1-RFP + NC85.2	NC105.1-RFP + NC85.2	NC105.1-RFP + NC85.2	NC105.1-RFP + NC85.2	NC105.1-RFP + NC85.2
Chimera/Control	Chimera	Chimera	Control	Control	Chimera
Time Point	16 h	8 h	20 h	12 h	20 h
Biological Replicate	2	1	2	1	2
Dye	Cy5	Cy5	Cy5	Cy5	Cy5
**Array 2**					
Experiment Set	NC105.1-RFP + NC85.2	NC105.1-RFP + NC85.2	NC105.1-RFP + NC85.2	NC105.1-RFP + NC85.2	NC105.1-RFP + NC85.2
Chimera/Control	Chimera	Control	Control	Chimera	Chimera
Time Point	12 h	4 h	16 h	4 h	16 h
Biological Replicate	1	2	1	2	1
Dye	Cy5	Cy5	Cy5	Cy5	Cy5
**Array 3**					
Experiment Set	NC105.1-RFP + NC85.2	NC105.1-RFP + NC85.2	NC105.1-RFP + NC85.2	NC105.1-RFP + NC85.2	NC105.1-RFP + NC85.2
Chimera/Control	Control	Control	Chimera	Chimera	Control
Time Point	8 h	20 h	8 h	20 h	12 h
Biological Replicate	2	1	2	1	2
Dye	Cy5	Cy5	Cy5	Cy5	Cy5
**Array 4**					
Experiment Set	NC105.1-RFP + NC85.2	NC105.1-RFP + NC85.2	NC105.1-RFP + NC85.2	NC105.1-RFP + NC85.2	NC105.1-RFP + NC85.2
Chimera/Control	Control	Chimera	Chimera	Control	Control
Time Point	4 h	12 h	4 h	16 h	8 h
Biological Replicate	1	2	1	2	1
Dye	Cy5	Cy5	Cy5	Cy5	Cy5
	**Slide 6**	**Slide 7**	**Slide 8**	**Slide 9**	**Slide 10**
**Array 1**					
Experiment Set	NC105.1-RFP + NC85.2	NC105.1-RFP + NC85.2	NC105.1-RFP + NC85.2	NC105.1-RFP + NC85.2	NC105.1-RFP + NC85.2
Chimera/Control	Chimera	Chimera	Control	Control	Chimera
Time Point	16 h	8 h	20 h	12 h	20 h
Biological Replicate	2	1	2	1	2
Dye	Cy3	Cy3	Cy3	Cy3	Cy3
**Array 2**					
Experiment Set	NC105.1-RFP + NC85.2	NC105.1-RFP + NC85.2	NC105.1-RFP + NC85.2	NC105.1-RFP + NC85.2	NC105.1-RFP + NC85.2
Chimera/Control	Chimera	Control	Control	Chimera	Chimera
Time Point	12 h	4 h	16 h	4 h	16 h
Biological Replicate	1	2	1	2	1
Dye	Cy3	Cy3	Cy3	Cy3	Cy3

**Array 3**					
Experiment Set	NC105.1-RFP + NC85.2	NC105.1-RFP + NC85.2	NC105.1-RFP + NC85.2	NC105.1-RFP + NC85.2	NC105.1-RFP + NC85.2
Chimera/Control	Control	Control	Chimera	Chimera	Control
Time Point	8 h	20 h	8 h	20 h	12 h
Biological Replicate	2	1	2	1	2
Dye	Cy3	Cy3	Cy3	Cy3	Cy3
**Array 4**					
Experiment Set	NC105.1-RFP + NC85.2	NC105.1-RFP + NC85.2	NC105.1-RFP + NC85.2	NC105.1-RFP + NC85.2	NC105.1-RFP + NC85.2
Chimera/Control	Control	Chimera	Chimera	Control	Control
Time Point	4 h	12 h	4 h	16 h	8 h
Biological Replicate	1	2	1	2	1
Dye	Cy3	Cy3	Cy3	Cy3	Cy3
	**Slide 11**	**Slide 12**	**Slide 13**	**Slide 14**	**Slide 15**
**Array 1**					
Experiment Set	NC105.1-RFP + NC28.1	NC105.1-RFP + NC28.1	NC105.1-RFP + NC28.1	NC105.1-RFP + NC28.1	NC105.1-RFP + NC28.1
Chimera/Control	Chimera	Chimera	Control	Control	Chimera
Time Point	16 h	8 h	20 h	12 h	20 h
Biological Replicate	2	1	2	1	2
Dye	Cy5	Cy5	Cy5	Cy5	Cy5
**Array 2**					
Experiment Set	NC105.1-RFP + NC28.1	NC105.1-RFP + NC28.1	NC105.1-RFP + NC28.1	NC105.1-RFP + NC28.1	NC105.1-RFP + NC28.1
Chimera/Control	Chimera	Control	Control	Chimera	Chimera
Time Point	12 h	4 h	16 h	4 h	16 h
Biological Replicate	1	2	1	2	1
Dye	Cy5	Cy5	Cy5	Cy5	Cy5
**Array 3**					
Experiment Set	NC105.1-RFP + NC28.1	NC105.1-RFP + NC28.1	NC105.1-RFP + NC28.1	NC105.1-RFP + NC28.1	NC105.1-RFP + NC28.1
Chimera/Control	Control	Control	Chimera	Chimera	Control
Time Point	8 h	20 h	8 h	20 h	12 h
Biological Replicate	2	1	2	1	2
Dye	Cy5	Cy5	Cy5	Cy5	Cy5
**Array 4**					
Experiment Set	NC105.1-RFP + NC28.1	NC105.1-RFP + NC28.1	NC105.1-RFP + NC28.1	NC105.1-RFP + NC28.1	NC105.1-RFP + NC28.1
Chimera/Control	Control	Chimera	Chimera	Control	Control
Time Point	4 h	12 h	4 h	16 h	8 h
Biological Replicate	1	2	1	2	1
Dye	Cy5	Cy5	Cy5	Cy5	Cy5
	**Slide 16**	**Slide 17**	**Slide 18**	**Slide 19**	**Slide 20**
**Array 1**					

Experiment Set	NC105.1-RFP + NC28.1	NC105.1-RFP + NC28.1	NC105.1-RFP + NC28.1	NC105.1-RFP + NC28.1	NC105.1-RFP + NC28.1
Chimera/Control	Chimera	Chimera	Control	Control	Chimera
Time Point	16 h	8 h	20 h	12 h	20 h
Biological Replicate	2	1	2	1	2
Dye	Cy3	Cy3	Cy3	Cy3	Cy3
**Array 2**					
Experiment Set	NC105.1-RFP + NC28.1	NC105.1-RFP + NC28.1	NC105.1-RFP + NC28.1	NC105.1-RFP + NC28.1	NC105.1-RFP + NC28.1
Chimera/Control	Chimera	Control	Control	Chimera	Chimera
Time Point	12 h	4 h	16 h	4 h	16 h
Biological Replicate	1	2	1	2	1
Dye	Cy3	Cy3	Cy3	Cy3	Cy3
**Array 3**					
Experiment Set	NC105.1-RFP + NC28.1	NC105.1-RFP + NC28.1	NC105.1-RFP + NC28.1	NC105.1-RFP + NC28.1	NC105.1-RFP + NC28.1
Chimera/Control	Control	Control	Chimera	Chimera	Control
Time Point	8 h	20 h	8 h	20 h	12 h
Biological Replicate	2	1	2	1	2
Dye	Cy3	Cy3	Cy3	Cy3	Cy3
**Array 4**					
Experiment Set	NC105.1-RFP + NC28.1	NC105.1-RFP + NC28.1	NC105.1-RFP + NC28.1	NC105.1-RFP + NC28.1	NC105.1-RFP + NC28.1
Chimera/Control	Control	Chimera	Chimera	Control	Control
Time Point	4 h	12 h	4 h	16 h	8 h
Biological Replicate	1	2	1	2	1
Dye	Cy3	Cy3	Cy3	Cy3	Cy3

	**Slide 21**	**Slide 22**	**Slide 23**	**Slide 24**	**Slide 25**
**Array 1**					
Experiment Set	NC105.1-RFP + NC63.2	NC105.1-RFP + NC63.2	NC105.1-RFP + NC63.2	NC105.1-RFP + NC63.2	NC105.1-RFP + NC63.2
Chimera/Control	Chimera	Chimera	Control	Control	Chimera
Time Point	16 h	8 h	20 h	12 h	20 h
Biological Replicate	2	1	2	1	2
Dye	Cy5	Cy5	Cy5	Cy5	Cy5
**Array 2**					
Experiment Set	NC105.1-RFP + NC63.2	NC105.1-RFP + NC63.2	NC105.1-RFP + NC63.2	NC105.1-RFP + NC63.2	NC105.1-RFP + NC63.2
Chimera/Control	Chimera	Control	Control	Chimera	Chimera
Time Point	12 h	4 h	16 h	4 h	16 h
Biological Replicate	1	2	1	2	1
Dye	Cy5	Cy5	Cy5	Cy5	Cy5
**Array 3**					
Experiment Set	NC105.1-RFP + NC63.2	NC105.1-RFP + NC63.2	NC105.1-RFP + NC63.2	NC105.1-RFP + NC63.2	NC105.1-RFP + NC63.2
Chimera/Control	Control	Control	Chimera	Chimera	Control

Time Point	8 h	20 h	8 h	20 h	12 h
Biological Replicate	2	1	2	1	2
Dye	Cy5	Cy5	Cy5	Cy5	Cy5
**Array 4**					
Experiment Set	NC105.1-RFP + NC63.2	NC105.1-RFP + NC63.2	NC105.1-RFP + NC63.2	NC105.1-RFP + NC63.2	NC105.1-RFP + NC63.2
Chimera/Control	Control	Chimera	Chimera	Control	Control
Time Point	4 h	12 h	4 h	16 h	8 h
Biological Replicate	1	2	1	2	1
Dye	Cy5	Cy5	Cy5	Cy5	Cy5
	**Slide 26**	**Slide 27**	**Slide 28**	**Slide 29**	**Slide 30**
**Array 1**					
Experiment Set	NC105.1-RFP + NC63.2	NC105.1-RFP + NC63.2	NC105.1-RFP + NC63.2	NC105.1-RFP + NC63.2	NC105.1-RFP + NC63.2
Chimera/Control	Chimera	Chimera	Control	Control	Chimera
Time Point	16 h	8 h	20 h	12 h	20 h
Biological Replicate	2	1	2	1	2
Dye	Cy3	Cy3	Cy3	Cy3	Cy3
**Array 2**					
Experiment Set	NC105.1-RFP + NC63.2	NC105.1-RFP + NC63.2	NC105.1-RFP + NC63.2	NC105.1-RFP + NC63.2	NC105.1-RFP + NC63.2
Chimera/Control	Chimera	Control	Control	Chimera	Chimera
Time Point	12 h	4 h	16 h	4 h	16 h
Biological Replicate	1	2	1	2	1
Dye	Cy3	Cy3	Cy3	Cy3	Cy3
**Array 3**					
Experiment Set	NC105.1-RFP + NC63.2	NC105.1-RFP + NC63.2	NC105.1-RFP + NC63.2	NC105.1-RFP + NC63.2	NC105.1-RFP + NC63.2
Chimera/Control	Control	Control	Chimera	Chimera	Control
Time Point	8 h	20 h	8 h	20 h	12 h
Biological Replicate	2	1	2	1	2
Dye	Cy3	Cy3	Cy3	Cy3	Cy3
**Array 4**					
Experiment Set	NC105.1-RFP + NC63.2	NC105.1-RFP + NC63.2	NC105.1-RFP + NC63.2	NC105.1-RFP + NC63.2	NC105.1-RFP + NC63.2
Chimera/Control	Control	Chimera	Chimera	Control	Control
Time Point	4 h	12 h	4 h	16 h	8 h
Biological Replicate	1	2	1	2	1
Dye	Cy3	Cy3	Cy3	Cy3	Cy3
